# Pneumatosis cystoides intestinalis associated with sunitinib and a literature review

**DOI:** 10.1186/s12885-017-3744-0

**Published:** 2017-11-09

**Authors:** Yong Suk Lee, Jae Joon Han, Si-Young Kim, Chi Hoon Maeng

**Affiliations:** Division of Medical Oncology-Hematology, Department of Internal Medicine, Kyung Hee University Hospital, College of Medicine, Kyung Hee University, (02447) 23 Kyungheedae-ro, Dongdaemun-gu, Seoul, South Korea

**Keywords:** Pneumatosis cystoides intestinalis, Sunitinib, Perforation

## Abstract

**Background:**

Pneumatosis cystoides intestinalis (PCI) is a rare self-limiting condition characterized by air-filled cysts within intestinal walls. Diagnosis should be prudent because it can mimic pneumoperitoneum leading to unnecessary treatment such as surgical exploration. Although various drugs including anti-neoplastic agents have been suggested as etiologies, cases related to sunitinib are sparse. Because of the rarity of this unusual side effect by sunitinib, we report the case report.

**Case presentation:**

A 68-year-old female with pancreatic neuroendocrine tumor who was treated with sunitinb for 4 months visited to our hospital complaining of severe diarrhea and mild abdominal discomfort. The abdominal X-ray showed subdiaphragmatic air mimicking intestinal perforation. After the meticulous evaluation including abdomino-pelvic computed tomography, the patient was diagnosed of PCI induced by sunitinib and fully recovered with conservative management.

**Conclusions:**

It is important to note that PCI can develop after treatment with sunitinib because PCI has not been widely known as an adverse event caused by the agent. Furthemore, emergent surgery while sunitinib was administrated without adequate washout period can result in substantial surgical complications which could be avoided with the precise diagnosis.

## Background

Pneumatosis cystoides intestinalis (PCI) is a rare condition characterized by air-filled cysts within intestinal walls. Although abdominal pain or distension can be associated with PCI, its symptoms are generally non-specific and can be incidentally identified by routine imaging study [1, 2]. PCI is categorized into either primary or secondary PCI. While primary PCI has an unknown etiology, various case reports of secondary PCI have suggested diverse causes [3]. Based on previous studies, physical causes such as intestinal obstruction or ischemia, pneumomediastinum extending to the abdominal cavity along with the great vessels, or infection could be associated with PCI. Anti-neoplastic agents have also been recently suggested as etiologic agents [1, 4].

Sunitinib is an oral multi-tyrosine kinase inhibitor targeting platelet-derived growth factor receptors (PDGFRα and PDGFRβ), vascular endothelial growth factor receptors (VEGFR1, VEGFR2, and VEGFR3), FMS-like tyrosine kinase-3 (FLT3), colony-stimulating factor type 1 (CSF-1R), and glial cell-line-derived neurotrophic factor receptor (RET). The anti-tumor and anti-angiogenic activity of sunitinib have led to its wide use at several types of cancer. Common adverse events of sunitinib include hypertension, diarrhea, nausea, asthenia, fatigue, vomiting, hand-foot syndrome, and hematologic toxicity [5, 6]. Herein, we report a rare case of PCI in a patient who was treated with sunitinib.

## Case presentation

A 68-year-old female with well-differentiated pancreatic neuroendocrine tumor visited an outpatient clinic due to persistent diarrhea. She had been previously found to have unresectable pancreatic neuroendocrine tumor with hepatic metastases. After disease progression despite prior therapy of long-acting octreotide analogue and everolimus, she had been treated with sunitinib as a third-line chemotherapy. After 3 months of sunitinib treatment, she showed partial response on follow-up abdominal computed tomography (CT) but complained of watery diarrhea. There was no definite cause of the diarrhea. Although it was partially controlled by loperamide, diarrhea persisted for over 1 month. Colonoscopy revealed no abnormal findings. Given the possibility of diarrhea due to adverse effects of sunitinib, the patient was treated with a reduced dose of sunitinib (25 mg/day) and loperamide concomitantly. After a brief period of improved diarrhea, however, she returned to the hospital complaining of severe diarrhea for over 1 week.

**Fig. 1:**
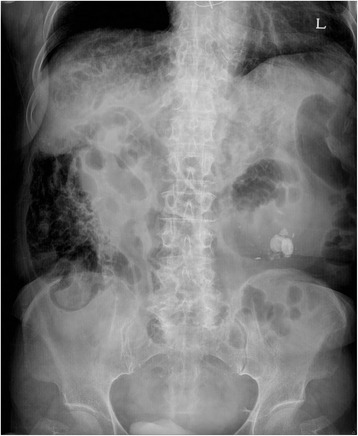
Erect view of abdominal X-ray at initial presentation

**Fig. 2:**
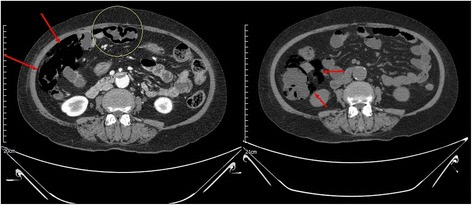
Abdominal CT at initial presentation. Note the air collection within ileal loops and colonic wall on initial CT (Left). PCI was severe, thus air-containing cysts (arrows and circle) were distributed at both of mesenteric and anti-mesenteric border. On the follow-up CT (Right) taken 1 week later, improving PCI was observed. Cysts at mesenteric border and near mesenteric vessels were predominant (arrows)

A simple chest X-ray taken on admission showed subdiaphragmatic air on the right side of the upper abdomen (Fig. 1) with severe distension. This was an unexpected finding because the patient did not complain of any signs of intestinal perforation, such as abdominal pain, tenderness, or hemodynamic instability. She complained of diarrhea, mild fatigue, dyspepsia, and vague abdominal discomfort. Her vital signs were stable as follows: blood pressure 140/90 mmHg, body temperature 36.6 °C, heart rate 78/min, respiratory rate 20/min. Blood tests showed no specific results. On physical examination, tympanic percussion on a distended abdomen and decreased bowel sounds were noted. Abdominal CT scan was performed to evaluate additional problems because her symptoms and signs were neither specific nor informative despite subdiaphragmatic air on chest X-ray. Abdominal CT scan showed diffuse air-filled cystic formation along with distal ileum and colon mimicking pneumoperitoneum (Fig. 2a, b). Although there was a large amount of air in the abdominal cavity on CT scan, it was along the bowel loop and confined to the intestinal wall rather than freely located. Divertucula were ruled out because the shape of air pocket was circular along with the luminal wall. Diverticulum is typically presented as a focal outpouching sac. Given the typical findings on abdominal CT, a diagnosis of PCI was made.

**Fig. 3:**
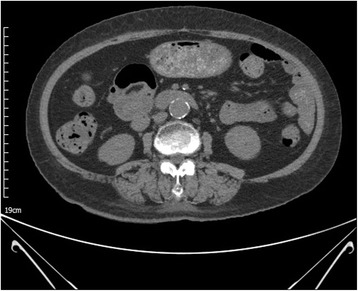
Follow-up abdominal CT after 2 week shows nearly resolved state of PCI

The patient received conservative management. Sunitinib was stopped since 1 day before admission and never reintroduced again. Supplemental oxygen was provided, and she was advised to avoid eating per os and provided with parenteral nutrition support for several days. Subsequent follow-up CT scans and abdominal X-ray showed improved gas contents within the bowel wall (Fig. 3). Two weeks later, she was completely recovered from PCI. Diarrhea was also improved a few days after discontinuation of sunitinib with conservative management such as hydration and loperamide.

## Discussion and conclusions

Although the pathogenesis has not been fully established, various causes or clinical situations have been suggested to explain the development of PCI. They are classified as the following categories [1, 2, 7–10]: (1) mechanical irritation or increased intra-abdominal pressure caused by surgery, trauma, or colonoscopy that causes intraluminal air to penetrate into the bowel wall; (2) respiratory disease such as chronic obstructive pulmonary disease can result in pneumomediastinum by increased pulmonary alveolar pressure and rupture, and the trapped air can move into the abdominal cavity; (3) bacterial overgrowth in the lumen can cause increased intraluminal gas and pressure to penetrate through a disrupted or damaged mucosal barrier; (4) Disequilibrium of luminal gas composition and pressure causing supersaturation of gas, and resultantly forming air bubbles in the wall along with the bowel vasculatures.

Recently, chemotherapeutic agents have been reported as the cause of PCI. A case report of PCI in a patient after one session of cytotoxic chemotherapy (daunorubicin, vincristine, L-asparaginase, and prednisolone as an induction treatment) explained that chemotherapy might increase the risk of infection and result in intestinal bacterial overgrowth [4]. The effect of gas-forming bacteria on the bowel wall can lead to air-filled cysts within the wall, increasing mucosal friability and permeability. Although there was no evidence of bacterial infection such as enterocolitis, repeated mucosal irritation could damage the bowel wall. According to previous studies, air-filled cysts within the bowel wall can develop by movement of intraluminal air into the wall after mucosal injury [1]. Relatedly, the submucosa is the most common site of PCI among the layers of the bowel wall [1]. The rate of all grades of diarrhea has been reported to be up to 60%, although diarrhea more severe than grade 3 develops less frequently (<10%) [6, 11]. Taken together, these findings indicate that severe diarrhea caused by sunitinib can be associated with mucosal irritation and damage that contributes to PCI.

An interesting and more relaible theory of PCI known as “Counterperfusion supersaturation” can be another explanation for the development of PCI in this case. [8–10]. Component of air within cysts in patients with PCI mainly composed of hydrogen, nitrogen, and carbon dioxide [12]. In normal circumstance, gas pressure of hydrogen produced by intestinal bacteria and nitrogen diffused from blood stream by pulmonary gas exchange reaches equilibrium. Under certain conditions such as excessive hydrogen production due to bacterial overgrowth, pressure gradient could be high enough to result in gas supersaturation, giving rise to gas-containing cystic formation under the influence of resistive index of colonic wall [8]. Although there was no clear evidence regarding this etiology associated with excessive hydrogen production including bacterial overgrowth, transient bowel inflammation with severe diarrhea might be related to such condition.

Furthermore, this theory gives another clue of relationship between PCI and its enhanced predisposition by antiangiogenic treatment. Sunitinib is a small molecule inhibitor mainly inhibiting VEGF receptors. Inhibition of angiogenesis may lead to decreased capillary density of the intestine. This could prevent efficient gas diffusion and exchange across the blood vessels and bowel lumen, leading to increase in gas pressure gradient predisposing gas supersaturation. Sunitinib treatment in our patient could be an explanation of possible diffusion barrier despite the fact that the patient did not have any medical history of vascular disease or chronic obstructive disease such as emphysema.

Besides, it is well known that anti-angiogenic therapy such as bevacizumab can result in gastrointestinal perforation [13]. In such situations, mucosal injury or microperforation of the wall could not be efficiently restored since blood supply via capillaries is disturbed [14]. Some authors have reported cases of sunitinib-induced intestinal perforation or PCI, associated with ischemic necrosis or pale-colored bowel [15, 16]. Since PCI can be accompanied by perforation, although it remains to be explained further whether PCI always precedes intestinal perforation, it is assumed that the mechanisms of PCI and intestinal perforation caused by anti-angiogenic agents including sunitinib are similar to each other [16, 17]. However, bowel perforation caused by PCI is not common because intraluminal air trapped in the mucosal or submucosal layer should penetrate all layers of the intestine to develop pneumoperitoneum.

Cases of PCI with or without intestinal perforation associated with anti-angiogenic therapy are summarized in Table 1. Most cases were diagnosed based on X-ray and abdominal CT scans, like our patient. In general, a diagnosis of PCI can be made by imaging rather than endoscopy or invasive biopsy [1]. Among the eight cases, surgical intervention was performed in three patients because of suspicious bowel perforation or necrosis. However, there was no case that actually showed acute ischemia or bowel necrosis consistent with bowel perforation and panperitonitis. After retrospectively reviewing the clinical courses of each report, surgical exploration is considered unnecessary to diagnose or treat cases of PCI. PCI improved only with conservative management or close observation. The majority of asymptomatic patients spontaneously improve without any intervention [1]. Even if patients have related symptoms, conservative management including fasting, parenteral nutrition, or intestinal decompression is sufficient, with excellent prognosis. Occasionally, portal venous gas can be noted on CT findings in cases of mesenteric ischemia, and surgery may be considered if frank bowel infarction is suspected [7]. Based on such clinical knowledge, our patient was closely observed with conservative management and recovered in 2 weeks. The time to recovery was consistent with the cases summarized in Table 1.

**Table 1:** Summarized case reports of PCI associated with anti-angiogenic therapy

Case Number (Reference)	Sex/Age	Site	Diagnostic tool	Underlying disease	Causative agent	Associated symptoms	Perforation	Treatment	Outcome	Time to recovery
1 [14]	M/54	Small intestine	CT	Pancreatic neuroendocrine tumor	S1 plus bevacizumab	Constipation	No	Observation	Resolved	6 weeks
2 [16]	F/Not described	Colon	CT, Biopsy	Renal cell carcinoma	Sunitinib	Flank pain and anuria	Yes	Operation	Resolved	NA
3 [16]	F/Not described	Colon	CT	Renal cell carcinoma	Sunitinib	Diarrhea and abdominal pain	Yes	Observation	Resolved	12 weeks
4 [17]	M/73	Colon	CT	Gastrointestinal stromal tumor	Sunitinib	None	Yes	Observation	Resolved	4 weeks
5 [18]	F/40	Small intestine	CT	Fibrolamellar carcinoma	Sorafenib	Abdominal pain, fever	Yes	Observation	Resolved	4 weeks
6 [18]	F/48	Small intestine	CT	Papillary thyroid carcinoma	Sunitinib	Diarrhea and abdominal pain	No	Observation	Resolved	4 weeks
7 [18]	M/68	Small intestine	CT	Renal cell carcinoma	Sunitinib	None	No	Operation	Resolved	NA
8 [15]	M/66	Small intestine	CT	Renal cell carcinoma	Sunitinib	Abdominal pain	None	Operation	Resolved	NA

There is an important point in terms of difference between PCI resulting from VEGF inhibitors and from other causes. Avoiding unnecessary surgical intervention is especially essential in PCI related to anti-angiogenic therapy. As shown in Table 1, surgical exploration is occasionally performed because PCI mimics bowel necrosis or panperitonitis. Since such an operation is performed in the emergency setting, an adequate washout period for anti-angiogenic therapy is not possible. This can lead to major postoperative co-morbidity such as delayed wound healing or hemorrhage.

In conclusion, with increasing use of VEGF inhibitors for various tumors, it is important to consider such uncommon adverse events because PCI was not mentioned as a drug-related adverse event in the previous clinical trials of these anti-neoplastic agents.

## Abbreviations

CSF-1R: Colony-stimulating factor type 1
CT: Computed tomography
FLT3: FMS-like tyrosine kinase-3
PCI: Pneumatosis cystoides intestinalis
PDGFR: Platelet-derived growth factor receptors
RET: Glial cell-line-derived neurotrophic factor receptor
VEGFR: Vascular endothelial growth factor receptors
